# Insights into the Potential of Sourdough-Related Lactic Acid Bacteria to Degrade Proteins in Wheat

**DOI:** 10.3390/microorganisms8111689

**Published:** 2020-10-30

**Authors:** Vera Fraberger, Martin Ladurner, Alexandra Nemec, Clemens Grunwald-Gruber, Lisa M. Call, Rupert Hochegger, Konrad J. Domig, Stefano D’Amico

**Affiliations:** 1 Department of Food Science and Technology, University of Natural Resources and Life Sciences Vienna (BOKU), 1190 Vienna, Austria; vera.fraberger@boku.ac.at (V.F.); ladurnermartin@students.boku.ac.at (M.L.); alexandra.nemec@students.boku.ac.at (A.N.); lisa.call@boku.ac.at (L.M.C.); konrad.domig@boku.ac.at (K.J.D.); 2 Department of Chemistry, University of Natural Resources and Life Sciences Vienna (BOKU), 1190 Vienna, Austria; clemens.gruber@boku.ac.at; 3 Institute for Animal Nutrition and Feed, Austrian Agency for Health and Food Safety (AGES), Spargelfeldstraße 191, 1220 Vienna, Austria; 4 Institute of Food Safety, Austrian Agency for Health and Food Safety (AGES), Spargelfeldstraße 191, 1220 Vienna, Austria; rupert.hochegger@ages.at

**Keywords:** sourdough, lactic acid bacteria, gluten, α-amylase-trypsin inhibitors, wheat sensitivity

## Abstract

Sourdough processing contributes to better digestible wheat-based bakery products, especially due to the proteolytic activity of lactic acid bacteria (LAB). Therefore, sourdough-related LAB were screened for their capacity to degrade immunogenic proteins like gluten and alpha-amylase-trypsin inhibitors (ATIs). Firstly, the growth of 87 isolates was evaluated on a gluten-based medium. Further, the breakdown capacity of selected isolates was determined for gluten with a focus on gliadins by measuring acidification parameters and MALDI-TOF MS protein profiles. ATI degradation after 72 h of incubation within an ATI-based medium was investigated by means of acidification, HPLC, and competitive ELISA. All isolates exhibited the potential to degrade ATIs to a high degree, whereas the gliadin degradation capacity varied more greatly among tested LAB, with *Lacticaseibacillus paracasei* Lpa4 exhibiting the strongest alterations of the gliadin pattern, followed by *Lactiplantibacillus plantarum* Lpl5. ATI degradation capacities ranged from 52.3% to 85.0% by HPLC and 22.2% to 70.2% by ELISA, with *Lacticaseibacillus paracasei* Lpa4 showing superior breakdown properties. Hence, a selection of specific starter cultures can be used in sourdough processing for wheat-based bakery products with reduced gluten and ATI content and, further, better tolerated products for patients suffering from non-celiac wheat sensitivity (NCWS).

## 1. Introduction

Wheat is a staple food worldwide, with a production of up to 760 million tons in September 2020 [1]. The increasing consumption of wheat and wheat-based products had led to an expansion of wheat-related disorders [2]. Diseases associated with wheat are celiac disease (CD) and IgE mediated allergies, with gluten as the main causative agent. This protein cooperates two fractions, namely gliadin (prolamin) and glutenin (glutelin). The gliadin fraction is further divided into α-, γ-, and ω-gliadins; glutenins are subdivided into high molecular weight (HMW) and low molecular weight (LMW) subunits [3]. Furthermore, it was reported that especially the α-gliadin family contains peptides exhibiting the highest immunogenic peptides, including a 33-mer peptide, p31–p43, a DQ2-restricted epitope, and a DQ8-restricted epitope [4].

A much higher percentage of people are estimated to exhibit symptoms after consuming gluten-containing food other than CD or wheat allergy, called non-celiac wheat sensitivity (NCWS). Due to the lack of reliable diagnostic markers, estimation is difficult. However, self-reported data led to a prevalence rate in the general population ranging from 0.5% to 13% [5]. Symptoms of NCWS include gastrointestinal (GI) symptoms like pain in the upper or lower abdomen, diarrhea, constipation, nausea, aphthous stomatitis, and altered bowel habits. However, even extra-intestinal symptoms have been reported, including lack of wellbeing, inability to concentrate, reduction of mnemonic capabilities, tiredness, headache, anxiety, numbness, joint/muscle pain, and skin rash [5]. The causative agents of NCWS are hitherto not clearly defined. However, the main triggers were reported to include FODMAPs (fermentable oligo-, di-, monosaccharides, and polyols) [6] and ATIs (alpha-amylase-trypsin inhibitors) [7,8]. The latter belong to the non-gluten wheat proteins, which lead to an intestinal innate immune response. The mechanism of the immune response has already been studied by Caminero et al. [9], showing the promotion of the TLR4-MD2-CD14 pathway, which resulted in barrier dysfunctions and intraepithelial lymphocytosis in mice when administering ATIs. Furthermore, in gluten-sensitive mice, ATIs were assumed to even increase the villus-to-crypt ratio alterations [9]. Further, a connection between the withdrawal of gluten and reduced symptoms in patients with IBS (irritable bowel syndrome) was observed [10].

ATIs and gluten are poorly digested by mammalian enzymes, but Caminero, et al. [9] already proved the reduced inflammatory effect of ATIs by administrating lactobacilli to mice as well as the degradation potential of lactic acid bacteria (LAB) isolated from the human gastrointestinal tract. However, pre-degradation of these proteins by fermentation might lead to better tolerated products. Sourdough processing has already been studied towards the potential to reduce compounds with adverse effects [11,12,13]. Especially the microbiota of sourdough, comprising yeasts and LAB, is responsible for health promoting effects. Furthermore, LAB which have been applied for decades in food preservation have been classified as Generally Recognized As Safe (G.R.A.S.), with some lactobacilli harboring the Qualified Presumption of Safety (QPS) status. Several studies already examined gluten hydrolysis by sourdough-related LAB [14,15,16,17,18], while Di Cagno, et al. [18] and Yin, et al. [17] additionally proved the degradation potential of sourdough LAB on water or salt soluble polypeptides, among which ATIs have a distinct share. Both studies revealed hydrolysis of non-gluten proteins by LAB, but no conclusion can be drawn due to missing assignments of ATIs.

Although the mentioned studies found proteolytic effects of LAB on wheat proteins, little information regarding the breakdown of ATIs is available. Only one study discovered the degradation of ATIs in sourdough bread [19]. However, to our knowledge, the degradation of ATIs as the sole protein sources for LAB has not been evaluated so far. Furthermore, the decomposition of gliadins as main contributors of immunogenic proteins in wheat-based bakery products was investigated. Therefore, this article describes the ability of sourdough-related LAB to degrade gliadins and ATIs.

## 2. Materials and Methods

### 2.1. Materials

Chemicals, solvents, and reagents were at least of p.A. grade, unless otherwise listed. For chromatographic and mass spectrometry, high-performance liquid chromatography (HPLC) gradient or liquid chromatography–mass spectrometry (LC-MS) grade solvents were used. Alpha-amylase inhibitor (AAI) from *Triticum aestivum* and soy trypsin-inhibitor standard was delivered from Sigma-Aldrich (USA). Wheat flour of Austrian type W700 was provided by the Pfahnl mill (Pregarten, Austria).

### 2.2. Isolation of Proteins

Gluten proteins were isolated by Glutomatic 2200 (Perten Instruments AB, Sweden) equipment according to ICC Standard No. 155. The gluten dough pieces were freeze dried at −50 °C and 0.57 mbar before being milled (1 mm sieve; IKA mill, Germany). The milled gluten powder was further used for gluten-based media (GBM).

For the alpha-amylase-trypsin inhibitor (ATI) extraction, firstly, the wheat flour was defatted by applying n-hexane (VWR International GmbH, Germany) in a ratio of 1:10. After vortexing and magnetic stirring (10 min, 400 rpm), defatted flour was separated from the hexane phase by centrifugation for 10 min at 4000 rpm. The supernatant was discarded, the defatting step repeated, and the defatted flour was dried overnight at 40 °C. Further, the defatted flour was extracted in 200 mL chloroform/methanol (CM) mixture at an equal ratio. After vortexing for 20 s at room temperature and magnetic stirring (10 min, 400 rpm), centrifugation for 10 min at 4000 rpm was applied. By using a Rotavapor (Büchi, Germany), the extraction solvent of the collected supernatants was further evaporated to dryness at about 250 mbar and 40 to 50 °C. The obtained solids were resuspended with 10 mL 0.9% sodium chloride solution to solubilize specifically ATIs. The ATI extract was centrifuged with settings described before. The final concentration was checked by measuring soluble proteins with the Bradford method according to Call, et al. [20].

### 2.3. Tested Isolates

Eighty-seven LAB isolates, previously isolated from traditional Austrian sourdoughs [21] or purchased from LMG (Belgian Coordination Collection of Microorganisms; Bruxelles, Belgium) or DSMZ (German Collection of Microorganisms and Cell Cultures, Braunschweig, Germany), were used for the screening of gluten degradation. Most of the examined species belonged to the former genus *Lactobacillus* that was recently re-evaluated regarding its taxonomy [22]. The majority of species analyzed were those commonly identified in sourdoughs: *Companilactobacillus (C.) kimchii*, *C. paralimentarius*, *Fructilactobacillus (F.) sanfranciscensis*, *Furfurilactobacillus (Fu.) rossiae*, *Lacticaseibacillus (Lac.) paracasei*, *Lactiplantibacillus (Lp.) paraplantarum*, *Lp. plantarum*, *Lactobacillus (L.) gallinarum*, *Loigolactobacillus (Lo.) coryniformis*, *Latilactobacillus (La.) curvatus*, *Paucilactobacillus (Pa.) xiangfangensis*, *Pa. vaccinostercus*, *Lentilactobacillus (Le.) diolivorans*, *Le. kisonensis*, *Le. otakiensis*, *Le. parabuchneri*, *Levilactobacillus (Lev.) hammesii*, *Lev. senmaizukei*, *Lev. spicheri*, *Lev. brevis*, *Limosilactobacillus (Li.) pontis*, *Leuconostoc (Lc.) citreum*, *Lc. mesenteroides*, *Lc. pseudomesenteroides*, *Pediococcus (P.) pentosaceus*, *Weissella (W.) cibaria*, and *W. viridescens*. Details of isolates used within this study are listed in Table S1 of the supplement. The isolates were propagated on MRS agar (Merck, Darmstadt, Germany) for 48 h at 30 °C under anaerobic conditions (N_2_ 85%, H_2_ 5%, CO_2_ 10%) using the MACS VA-500 anaerobic incubator (Don Whitley Scientific, West Yorkshire, UK). When used for screening tests, LAB cells were incubated for 24 h in MRS broth (Merck, Darmstadt, Germany).

### 2.4. Growth Screening on a Gluten-Based Agar

For the preliminary screening of the proteolytic activity, a gluten-based agar (GBA) according to Gerez, et al. [16] was used with some modifications. Briefly, the medium was prepared with distilled water comprising (*w*/*v*) 4.5% gluten, 2% glucose, 1% KH_2_PO_4_, 1% K_2_HPO_4_ (Sigma-Aldrich, St. Louis, MO, USA), and 10% agar (VWR International GmbH, Darmstadt, Germany). Twenty microliters of cell suspensions of each LAB isolate (8 to 9 log_10_ CFU g^−1^) were dropped on GBA, and plates were incubated at 30 °C for up to 72 h.

### 2.5. Gliadin Degradation Capacity

Isolates showing the strongest proteolytic activity on GBA were further used for quantification analysis. Hence, 9 LAB isolates, *Lev. brevis* Lb3, *Lo. coryniformis* Lco4, *La. curvatus* Luc4, *Lac. paracasei* Lpa4, *Lp. plantarum* Lpl5 and Lpl7, *Lc. mesenteroides* Lm3, *Lc. pseudomesenteroides* Leps1, and *P. pentosaceus* Pp3, were further used to determine the degradation capacity regarding gluten proteins with a focus on gliadins. Therefore, cells cultivated in 10 mL MRS broth were harvested and washed using 10 mL PBS (phosphate-buffered saline; Sigma-Aldrich, St. Louis, MO, USA) by centrifugation at 4 °C, 8000× *g* for 6 min and resuspended in 10 mL sterile water. GBM (pH 6.67) was inoculated with the corresponding cell suspension (1% *v*/*v*) and incubated while continuously shaking for 72 h at 30 °C under anaerobic conditions. A homogenous suspension of GBM was achieved with an ultraturrax device (T-25 from IKA; Staufen, Germany).

Water soluble degradation products, mainly peptides, and gliadins were extracted by Osborne fractionation according to Call, et al. [23] with minor modifications. Instead of a sodium-chloride solution, water was used for the first extraction step. The supernatant of each fraction was transferred into 5 mL volumetric flasks, filter sterilized (0.45 µm; Phenomenex, Germany), and stored at −20 °C until further analysis.

Measurements of the pH were conducted after 72 h of incubation using a pH meter Lab 854 equipped with an N6000 BNC electrode (SI Analytics, Germany). Organic acids (lactic and acetic acid) were analyzed using an IEC dual analysis system ICS-5000 (Dionex, Sunnyvale, CA, USA), equipped with an AMINEX HPX-87H analytical column (300 mm × 7.8 mm). Identification and quantification of lactic and acetic acid was carried out using a UV–VIS detector (UDV 170U; Dionex, Sunnyvale, CA, USA) set at 210 nm according to Bender, et al. [24].

### 2.6. Growth Determination on ATI-Based Medium

Isolates as described in Section 2.5. were further analyzed regarding their ATI degradation potential. Furthermore, the isolate *F. sanfranciscensis* Lsa3 was added to the selection due to its dominance in type I sourdoughs [25]. Turbidity data for growth curves of LAB isolates were determined using the automated density monitoring system BioscreenC analyze reader (Oy Growth Curves Ab Ltd., Helsinki, Finland). The sterile filtered ATI-medium containing 0.21% ATI isolate, 2% glucose, 1% KH_2_PO_4_, and 1% K_2_HPO_4_ was inoculated (1% *v*/*v*) with each LAB isolate, exhibiting cell counts ranging from 8 to 9 log_10_ CFU g^−1^, and overlaid with mineral oil to obtain anaerobic conditions. Reading for 96 h at 30 °C, measurements were taken at an optical density of 600 nm (OD_600_) every 15 min after shaking.

ATI hydrolyzation was further determined within a model system after 72 h of incubation. The respective cell mass was transferred into centrifugation tubes and centrifuged at 8000× *g* for 6 min at 4 °C. The supernatant was further transferred to a volumetric flask and filled up to 1 mL. Next, the solution was filtered through a 0.45 µm filter and transferred to vials (1.5 mL clear glass) for further analysis.

pH values were determined after 72 h of incubation using an N6000 BNC electrode. Organic acid production was measured after 72 h with the lactic acid and acetic acid kit for the RIDA^®^CUBE scan system (R-Biopharm, Darmstadt, Germany) according to the manufacturers’ instructions.

### 2.7. Protein Analysis and Determination of Free Amino Acids

Protein profiling and molecular weight determination was performed by MALDI-TOF mass spectrometry (Microflex system, Bruker, Bremen, Germany). Extracted proteins were purified and enriched by SPME with C18 ZipTips (10 µL volume; Merck, Darmstadt, Germany). The clean-up procedure followed the instructions of the manufacturer. A final volume of about 1 µL was directly eluted to steel plates (MSP 96; Bruker, Bremen, Germany). The dried droplet was covered with matrix containing 10 mg/mL sinapic acid in a water–acetonitrile mixture containing 0.1% TFA. Spectra were generated with 100% laser energy and pulsed ion extraction set to 1000 ns for proteins in the range of 10 to 25 kDa or 150 ns for peptides in the range from 1 to 10 kDa. At least 5 single spectra generated by 200 laser shots were summarized for each sample.

Protein identification of the ATI isolate was executed by peptide mass fingerprints (PMF) after tryptic digestion followed by LC-HR-QTOF-MS and comparison of detected peptides with MASCOT software according to Call, et al. [20]. Briefly, in solution digestion of the ATI extract with trypsin was performed after reduction and alkylation with iodoacetamide. Peptide masses were obtained by separation with nano-LC system and detection with Bruker maxis 4G Q-TOF MS. Peptide masses in MS and MS2 were used for identification with MASCOT for IDs.

For quantification of ATIs by RP-HPLC, a Chromaster Series 5000 HPLC System from Hitachi with UV detection at 214 nm was used. Separation was performed with gradient elution (A: water with 0.1% TFA; B: acetonitrile with 0.1% TFA) on a HALO C18 wide pore column (150 mm × 2.1 mm, 2.7 µm particle size and 1000 Å). External calibration with trypsin inhibitor from soy according to Call, et al. [23] was applied. Furthermore, quantification of ATI reduction via competitive ELISA measurements (RIDASCREEN Gliadin competitive; R-Biopharm, Darmstadt, Germany) was applied according to the manufacturers’ instructions. The cubic spline fitting procedure was used for calibration and quantification with RIDASOFT Win Z9999 software (R-Biopharm, Darmstadt, Germany). Samples were analyzed in duplicate, with three repetitions each.

To provide information about the proteolytic activity, free amino acids were determined. Measurements took place after 72 h of incubation using the obtained filtered solutions as described in Section 2.5 to determine the gluten degradation capacity as well as Section 2.6 to analyze the ATI breakdown. The standard FAN (free amino nitrogen) assay at pH 6.8, as previously described by Belina-Aldemita, et al. [26], was applied. Glycine was used as standard for calibration and results are given in glycine equivalents.

### 2.8. Statistics

Correlation analysis according to Pearson and ANOVA was performed with SPSS software version 26 (IBM, USA). For correlation analysis, bivariate correlations according to Pearson were used, with a *p*-level ≤ 0.05 considered as significant and *p*-values of ≤0.01 as highly significant. The Scheffe procedure was used as post-hoc test.

## 3. Results and Discussion

### 3.1. Protein Extraction and Characterization

The extraction of wet gluten according to ICC Standard No. 155 resulted in a high purity isolate with 72.43% ± 0.80% protein. The wet gluten was used after freezing and milling as sole protein in a gluten-based medium (GBM), which was sterilized at 115 °C before usage. As expected, the heat impact altered the protein profile of gliadins (results not shown). Due to the low solubility in water, gluten proteins were suspended by high shear forces with an ultraturrax.

**Figure 1:**
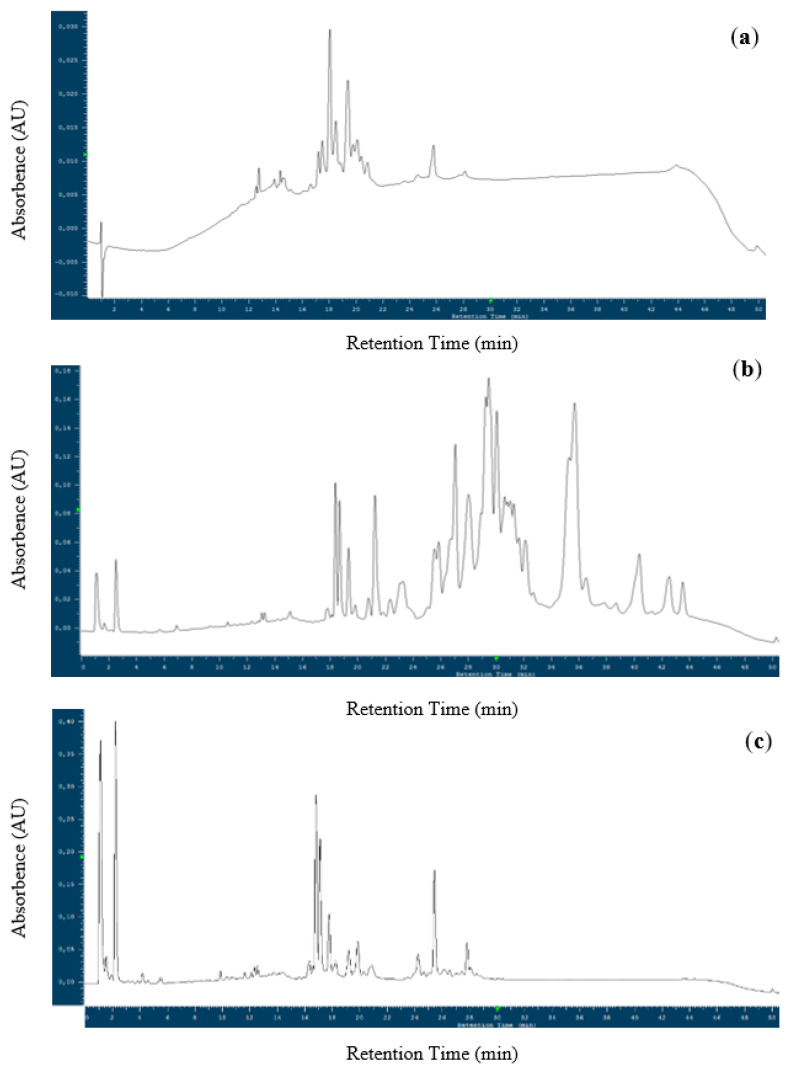
HPLC profiles of alpha-amylase inhibitor standard (AAI; Sigma-Aldrich, USA) (**a**), intermediate of chloroform methanol (CM) extraction (**b**), and final amylase trypsin inhibitor (ATI) extract (**c**).

The two-step alpha-amylase-trypsin inhibitors (ATIs) extraction procedure was able to isolate ATIs with low abundance of other proteins. Gliadins were effectively removed by resuspending the dried crude extract in sodium chloride solution. The obtained ATI extract showed a concentration of approximately 2.1 mg/mL protein, which was directly used for further growth experiments due to the high solubility. Figure 1 illustrates the RP-HPLC chromatograms of the final isolate (Figure 1c) and the intermediate extract (crude CM extract; Figure 1b). For comparison, the alpha-amylase inhibitor (AAI) standard from wheat was analyzed as well (Figure 1a). Spectra confirmed that the isolate predominately contained ATIs, as peaks showed the same retention times as the AAI standard. However, previous studies revealed low purity of the AAI standard of approximately 50% [20,27]. Thus, protein identification was performed by tryptic digestion and detection of peptides via LC-HR-QTOF MS, which were sent to the MASCOT search engine. As listed in Table S2, predominately ATIs with high scores and sequence coverages were identified. Monomeric (0.28) and dimeric (0.19 and 0.53) AAIs dominated, as seen in the highest scores, followed by CM3, CMd, and CM2. Furthermore, subtilisin-chymotrypsin and Bowman-Birk-type proteinase inhibitors were found. The identified ATIs in common bread wheat were in accordance with other studies [27,28]. Besides the mentioned inhibitors, some other proteins were detected as well, namely avenin-like proteins (farinins), non-specific lipid transfer protein, and purothionins. These proteins showed mainly much lower scores and sequence coverages, which indicated a lower abundance. The obtained identifications were confirmed by MALDI-TOF MS of whole proteins (Figure S1). Detected masses were mainly in the range of 12 to 16 kDa, which can be assigned to ATIs as determined by previous studies [20,23,29]. Again, the absence of gliadins with masses above 30 kDa was evident for the ATI isolate (Figure S1b), whereas gliadins exhibited a strong abundance in the intermediate extract (see Figure S1a). Due to the fact that all these proteins belong to the prolamin superfamily, having similar properties (molecular weight, hydrophobicity, immunogenic potential) [30], further purification seems to be very complicated and time consuming. Summing up, the produced extract contained highly concentrated ATIs with low concentrations of other proteins, which was confirmed by three different methods.

### 3.2. Gluten Degradation Capacity of LAB

The screening of 87 lactic acid bacteria (LAB) isolates on a cultivation medium with gluten (GBM) as the only nitrogen source (Figure S2) revealed that of the tested isolates, 4% exhibited no, 52% intermediate, and 18% strong growth on GBM. Table S1 lists LAB and their corresponding growth. These results even showed strain-dependent proteolytic activity, as, for example, *Le. parabuchneri* Lpb3 and *Li. pontis* Lpo1 exhibited no growth, compared to *Le. parabuchneri* isolates Lpb1, Lpb2, Lpb4, and *Li. pontis* Lpo2. This was even observed by Vermeulen, et al. [31] for *F. sanfranciscensis*. Evaluated proteolytic activity among LAB isolates was in accordance with other results [32]. Hence, the acquired results were suitable for the selection of samples for further experiments, with nine isolates (*Lev. brevis* Lb3, *Lo. coryniformis* Lco4, *La. curvatus* Luc4, *Lac. paracasei* Lpa4, *Lp. plantarum* Lpl5 and Lpl7, *Lc. mesenteroides* Lm3, *Lc. pseudomesenteroides* Leps1, *P. pentosaceus* Pp3) being selected.

#### 3.2.1. Determination of Gluten Degradation by MALDI-TOF MS

**Figure 2:**
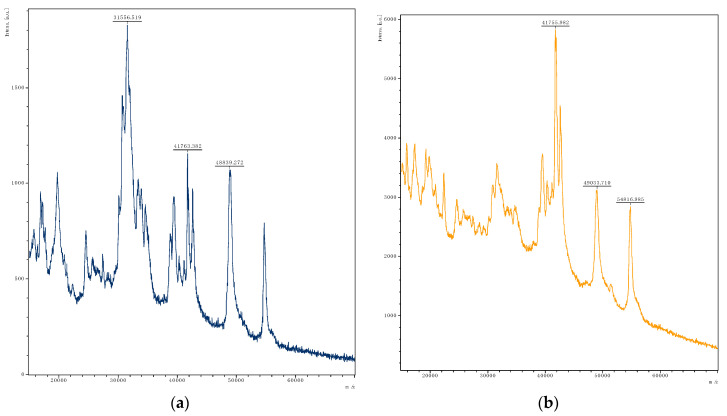

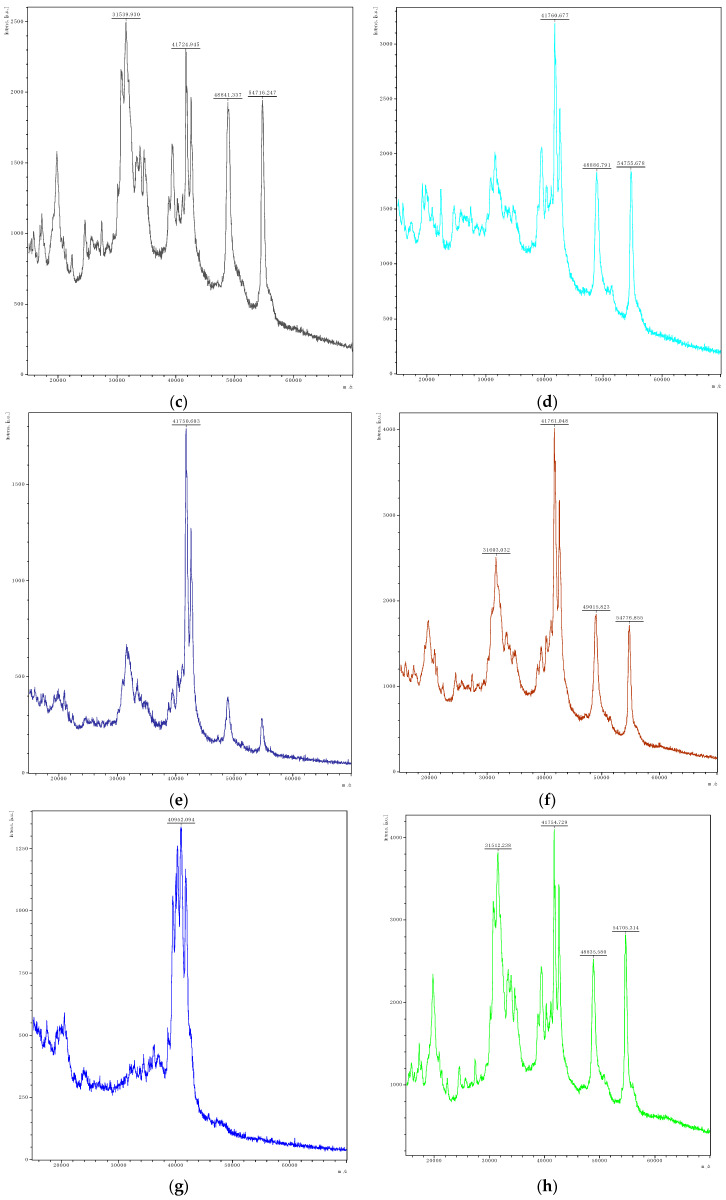

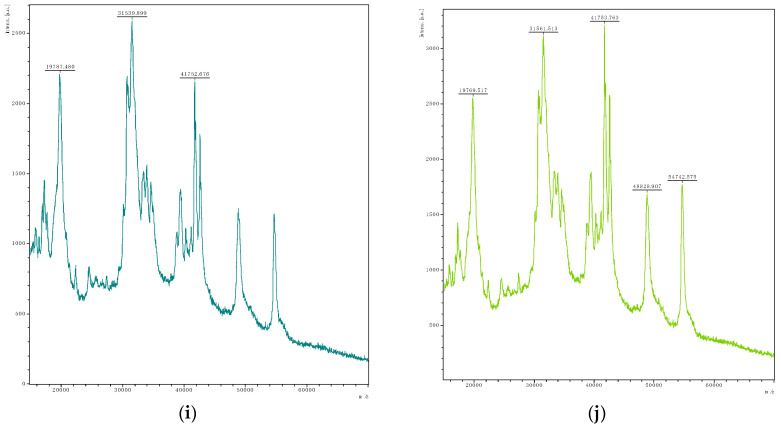
MALDI-TOF MS spectra of gliadins in the mass to charge (m/z) range of 15 to 70 kDa of (**a**) reference and after the degradation (72 h, 30 °C) by (**b**) *Levilactobacillus brevis* Lb3, (**c**) *Latilactobacillus curvatus* Lcu4, (**d**) *Loigolactobacillus coryniformis* Lco4, (**e**) *Lactiplantibacillus plantarum* Lpl5, (**f**) *Lp. plantarum* Lpl7, (**g**) *Lacticaseibacillus paracasei* Lpa4, (**h**) *Leuconostoc pseudomesenteroides* Leps1, (**i**) *Pediococcus pentosaceus* Pp3, (**j**) *Lc. mesenteroides* Lm3.

The reference without incubation with LAB (Figure 2a) showed a typical gliadin pattern with dominant peaks from 30 to 40 kDa, but some alterations were found as well. High peaks at about 49 and 55 kDa were detected, which can be assigned ω-gliadins. Usually, the ω-gliadins occur in minor concentrations compared to other gliadins and produce low intensity signals by MALDI-TOF MS, as seen by De Angelis, et al. [33]. Probably, the thermal impact could have altered single gliadin proteins. Furthermore, signals with moderate intensities below 30 kDa were visible. Obtained MALDI-TOF MS spectra of gliadins (Figure 2) showed the strongest decomposition of gliadins by *Lac. paracasei* Lpa4 (Figure 2g); only a few proteins with masses around 40 kDa remained after incubation. *Lp. plantarum* Lpl5 gained strong disintegration as well, but residual signals around 30 to 38, 42, 49, and 55 kDa were still present (Figure 2e). A similar hydrolysis pattern but higher intensities were observed for *Lev. brevis* Lb3 (Figure 2b), *Lo. coryniformis* (Figure 2d), and *Lp. plantarum* Lpl7 (Figure 2f). Moderate degradation of proteins was visible among fractions between 20 and 32 kDa for *L. brevis* Lb3, *Lo. coryniformis* Lco4, and *Lp. plantarum* Lpl7. Furthermore, due to the change in the relative intensities even for *Lc*. *pseudomesenteroids* Leps1, a degradation capacity of gliadins between 30 and 38 kDa was evident. *P. pentosaceus* Pp3 and *Lc. mesenteroides* Lm3 exhibited the lowest potential to degrade gliadins as no distinct changes within the peak pattern between 30 and 60 kDa were observable. Only a reduction in relative intensities in respect to the highest gliadin peak at about 32 kDa was detectable. However, due to the increased relative intensity of the peak at 20 kDa, it can be concluded that the presence of smaller protein fragments increased after 72 h of incubation.

MALDI-TOF MS spectra of water-soluble degradation products (Figure S3) revealed that *Lev. brevis* Lb3, *Lo. coryniformis* Lco4, and *Lp. plantarum* Lpl5 and Lpl7 exhibited only limited potential to hydrolyze gliadins to smaller peptides. *La. curvatus* Lcu4 showed an intermediate capacity, whereas *Lac. paracasei* Lpa4, *Lc. pseudomesenteroides* Leps1, *P. pentosaceus* Pp3, and *Lc. mesenteroides* Lm3 showed increased decomposition to peptides. For those isolates exhibiting the highest capacity, even the strongest rise in new peptides was observed compared to the reference. Further, a sharp rise in the baseline was detected, which indicated an increased number of degradation products. Especially fragments with a size of approximately 1.5 kDa were produced by *Lac. paracasei* Lpa4 and *Lc. pseudomesenteroides* Leps1. Analysis of both extracts showed only moderate consistency; not all isolates with strong gliadin degradation exhibited increased production of peptides. This discrepancy can be explained by the metabolization of peptides by LAB used for their growth, or by the occurrence of metabolites with higher masses not detected by the used settings (from 4 to 15 kDa).

Already, Gerez, et al. [34] have determined the gliadin degradation potential of LAB, showing an increased capacity by *Lp. plantarum*. Further, Dallagnol, et al. [35] examined the degradation potential of the strain *Lp. plantarum* CRL 778 on different protein fractions including gliadins and glutenins, showing a decrease in these fractions to different extents after 24 h. Within this study, the gliadin degradation by the tested isolates might be underestimated as the solubility of LMW glutenins in aqueous alcohol increases during fermentation, as described by Loponen, et al. [36]. Complete decomposition of gliadins as shown by Alvarez-Sieiro, et al. [32] can be only achieved by the combination of multiple strains. Furthermore, autoclaving of the GBM had an impact on the protein pattern. When comparing the obtained spectrum of the reference gliadin spectra (Figure 2a) with data from Alvarez-Sieiro, et al. [32], peaks greater than 40 kDa with increased intensities were observable for the heated gluten medium. Furthermore, even the comparison of RP-HPLC spectra of native gliadins and gliadins after autoclaving showed adulterations due to the thermal impact (results not shown).

#### 3.2.2. Determination of Acidification Parameters

Determination of the growth performance of selected isolates in liquid GBM as illustrated in Table 1 showed the strongest pH decrease by isolates *Lp. plantarum* Lpl5 and Lpl7 and *Lac. paracasei* Lpa4. Further, these isolates exhibited the highest production of lactic acid, with *Lp. plantarum* Lpl5 producing 2.79 g/L, followed by *Lp. plantarum* Lpl7 with 2.44 g/L, and *Lac. paracasei* Lpa4 with 2.39 g/L, respectively. Acetic acid concentrations remained low, with *Lc. mesenteroides* Lm3 exhibiting the highest production capacity of 0.18 mg/L. These results were in accordance with those obtained by Gerez, et al. [16] as *Lp. plantarum* isolates even showed the highest lactic acid production capacity compared to other tested LAB growing in GBM. The highest FAN content after fermentation was observable for *Lac. paracasei* Lpa4 (55.67 mg glycine/L). Further isolates exhibited approximately 10 times lower counts.

### 3.3. Degradation of ATIs by LAB

To test whether LAB can reduce the ATI concentration in an ATI-based medium, nine LAB isolates exhibiting a substantial ability to grow on gluten-based agar as well as *F. sanfranciscensis* Lsa3, the typical traditional sourdough-related species, were selected.

#### 3.3.1. Growth Curve Determination

**Figure 3:**
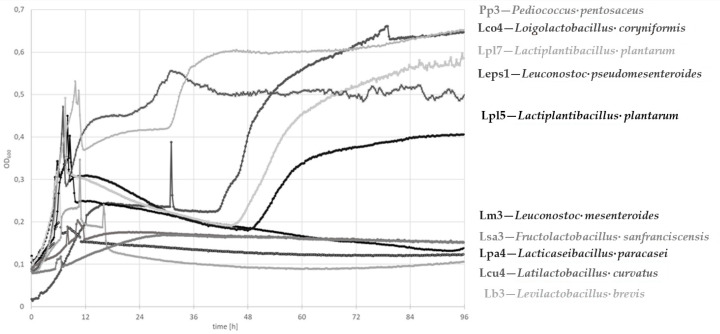
Growth curve determination by OD_600_ measurements for up to 96 h at 30 °C.

The growth curves measured at an optical density of 600 nm (OD_600_), as displayed in Figure 3, revealed the highest increase by *P. pentosaceus* Pp3 and *Lo. coryniformis* Lco4, followed by *Lp. plantarum* Lpl7 and *Lc. pseudomesenteroides* Leps1. *F. sanfranciscensis* Lsa3, *Lc. mesenteroides* Lm3, *Lac. paracasei* Lpa4, *La. curvatus* Lcu4, and *Lev. brevis* Lb3 only exhibited limited increases in the OD_600_. At the beginning of the growth curve, often some kind of sudden signal rise, similar to peaks, was observed only with respect to *Lo. coryniformis*, which showed this sudden increase after an incubation of about 30 h. Probably, the lowered pH due to acidification matched a point where proteins lost their charged state at the isoelectric point (IEP) and the high solubility in water-based solvents. Afterwards, the pH was further lowered and the region of IEP was passed, which resulted in the disappearance of these signals and realignment of curves. This explanation is in accordance with the IEPs of ATIs, which are mainly between 6.14 and 6.77 [28].

#### 3.3.2. Determination of Acidification Parameters

Concentrations of organic acids and final pH after fermentation for 72 h are listed in Table 2. The decrease in the pH was highest for *Lac. paracasei* Lpa4, followed by *Lp. plantarum* Lpl7 and *Lc. pseudomesenteroides* Leps1. These isolates even showed the highest production rates of lactic acid, exceeding 1250 mg/L. Acetic acid concentrations were lower compared to lactic acid content, with a maximum level being observed for *Lc. pseudomesenteroides* Leps1. Especially under anaerobic conditions, the predominant release of lactic acid compared to acetic acid remains the major product of metabolism for most LAB [37]. Mainly, high consistency between acidification on gluten and ATI based medium was found, except for *Lo. coryniformis* Lco4 and *Lc. pseudomesenteroides* Leps1, which did not show a decrease in the pH in the GBM. Furthermore, no relationship between pH and the increase in the OD_600_ growth was observed for ATIs.

Due to the hydrolysis of proteins by LAB during fermentation, free amino nitrogen (FAN) concentrations increase [38]. Within our study, results ranged from about 8 to 43 mg/L, with *Lo. coryniformis* Lco4 exhibiting the lowest levels. All other isolates produced distinctly more FAN, with concentrations above 20 mg/L. Furthermore, higher FAN values were observed for isolates exhibiting less acidic release, which was even observed by Filannino, et al. [39]. In general, highly significant correlations between the outcomes of the FAN analysis and pH (R^2^ 0.664 and *p* ≤ 0.01), as well as FAN and lactic acid results, were observable (R^2^ −0.663 and *p* ≤ 0.01).

However, due to the amino acid metabolism by LAB, FAN can be converted into a number of further compounds, resulting in reduced FAN concentrations, as already reported by Tavaria, et al. [40]. Generally, the FAN analysis was used to monitor the degree of the overall proteolysis, with a focus on exopeptidase activity [41]. Thus, proteolysis might be underestimated due to the amino acid metabolism exerted by LAB.

#### 3.3.3. Determination of the Degradation Capacity

**Figure 4:**
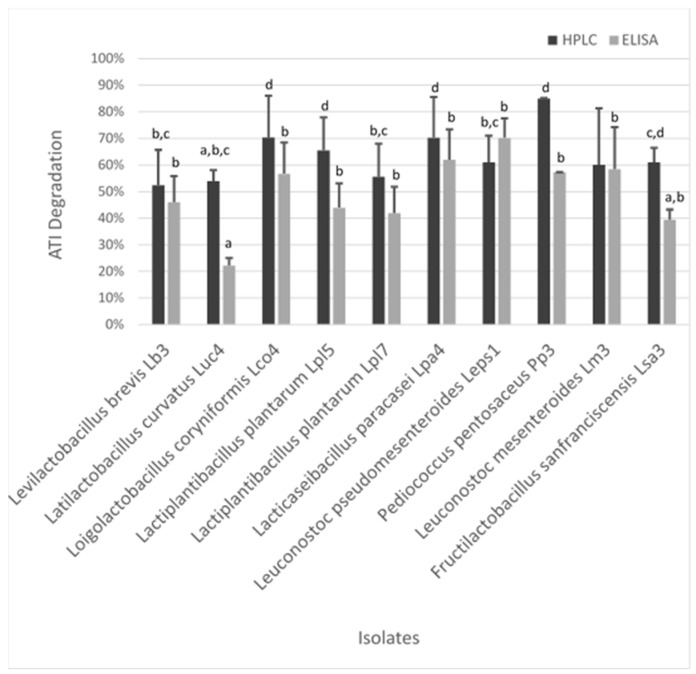
ATI hydrolysis capacity (%) of sourdough-related lactic acid bacteria measured by RP-HPLC and competitive ELISA (*n* = 6) after 72 h of incubation at 30 °C; small letters indicate homogenous subgroups based on ANOVA (*p* ≤ 0.05) and post-hoc test according to Scheffe.

To quantify the degradation capacity of LAB in respect to ATI isolates, HPLC and competitive ELISA measurements were conducted, with results being illustrated in Figure 4. Degradation capacity measured by HPLC ranged from 52.3% to 85.0%. *P. pentosaceus* Pp3, which displayed even the highest increase in the OD_600_ value and FAN content, showed the greatest hydrolyzation potential, followed by *Lo. coryniformis* Lco4 (70.3%) and *Lac. paracasei* Lpa4 (70.2%). Isolates of *Lp. plantarum* exhibited different degradation capacities, revealing a strain-dependent effect, with Lpl5 having significantly greater potential (65.5%) compared to Lpl7 (55.5%). Furthermore, lactic acid concentrations were increased and pH values lowered for Lpl7 in contrast to Lpl5. *Lc. pseudomesenteroides* Leps1 and *F. sanfranciscensis* Lsa3 showed a similar extent of degradation, followed by *Lc. mesenteroides* Lm3 with around 60%. However, lactic acid concentrations for Lsa3 were below the detection limit (<35 mg/L). With a reduction of 52.3%, *Lev. brevis* Lb3 exhibited the lowest capacity. Generally, the determined differences were mainly not significant due to a quite high standard deviation.

Results obtained by competitive ELISA measurements revealed a breakdown of ATIs ranging from 22.2% to 70.2%, which showed a similar picture as illustrated by the HPLC results. *Lc. pseudomesenteroides* Leps1 exhibited the highest capacity, followed by *Lac. paracasei* Lpa4. Furthermore, both showed high lactic acid concentrations. Further, *Lc. mesenteroides* Lm3, *P. pentosaceus* Pp3, and *Lo. coryniformis* Lco4 degraded less than 60% of present ATIs. Only a share of less than 50% of ATIs were metabolized by *Lev. brevis* Lb3, *Lp. plantarum* Lpl7 and Lpl5, and *F. sanfranciscensis* Lsa3. The lowest degradation potential on a significant level was observed for *La. curvatus* Lcu4.

Generally, high standard deviations were obtained for RP-HPLC and ELISA measurements, which can be explained by the low sample volume of only 200 µL and the moderate robustness of the single methods, especially concerning ELISA. Results from RP-HPLC revealed stronger hydrolysis compared to ELISA, except for Leps1. These circumstances can be explained by the different detection mechanisms. Nevertheless, a highly significant correlation of R^2^ 0.740 (*p* ≤ 0.01) between ATI degradation capacity determined by HPLC and ELISA response was obtained, which revealed the high consistency of both analytical methods. Further, no significant correlation between acidification parameters or growth rate and ATI reduction was observed; thus, a direct relationship can be excluded. At first glance, high consistency between hydrolysis and production of amino acids was noticeable as *P. pentosaceus* Pp3 showed superior ATI degradation and concentration of amino acids. Nevertheless, other isolates did not show such uniformity, which did not result in a significant relationship.

This study discovered a low response of ATIs to the R5 antibody commonly used for gliadin detection by competitive ELISA measurements, which is preferred for analysis of fermented foods. These findings are controversial due to the general status of non-gluten proteins regarding the immunogenic potential towards celiac disease (CD). However, already, Sanchez, et al. [42] have observed an antibody response to alpha-amylase inhibitor 0.19 by patients with celiac disease. Furthermore, Huebener, et al. [43] demonstrated a strong humoral response of CD patients to several non-gluten proteins of wheat. Identification of two-dimensionally separated proteins showed the high immunoreactive potential of ATIs. Probably, short overlapping sequences with certain γ-gliadin and LMW glutenins may have contributed to these outcomes [43]. Although analysis of PMF verified the absence of gluten proteins, marginal concentrations of gliadins could be overlooked due to the poor digestive performance of trypsin in respect to gluten proteins [44]. However, as already discussed [43], ATIs could act as antibody target proteins for CD, but more studies have to be conducted to clarify the role of ATIs in respect to CD.

These results showed the capacity of sourdough-related LAB to degrade ATIs in a model system. Already, Laatikainen, et al. [19] determined a reduction in the ATI content in sourdough bread; however, no information on the microbiota of sourdough was provided. Previous studies extensively revealed the potential of LAB to degrade protein fractions with a focus on gluten [15,17,18,42,45], whereas a few of these determined the degradation of proteins belonging to albumins/globulins as well. Although these studies did not explicitly determine ATIs, the albumin/globulin fractions studied even contained ATIs, as these are salt-soluble proteins. As determined by Yin, et al. [17] and Di Cagno, et al. [18], salt-soluble proteins with a size of less than 18 kDa were degraded during fermentation applying different lactobacilli as starter culture. Although not identified, these proteins can be assigned mainly to ATIs due to their molecular weights [20,43]. Within the study performed by Di Cagno, et al. [18], *Lev. brevis* exhibited the highest potential to degrade proteins in the range of 15 kDa. Further, Yin, et al. [17] proved the degradation of proteins within this range during sourdough fermentation with *Lp. plantarum* as starter culture. Caminero, et al. [9] confirmed the potential of LAB isolated from the human gut to degrade ATIs. Furthermore, strain-dependent performance was observed.

## 4. Conclusions

Within this study, the degradation potential of sourdough-related lactic acid bacteria (LAB) towards immunogenic wheat proteins with a focus on amylase-trypsin inhibitors (ATIs) and gliadins was investigated. Both cultivation media applied within this work were appropriate for the preselection of LAB, to examine gliadin conversion and to screen the ATI degradation potential. MALDI-TOF MS was a fast and suitable tool to monitor gliadin hydrolysis by alteration of protein patterns. Quantification of proteins conducted by RP-HPLC and ELISA was able to evaluate the decomposition of ATIs. However, further examinations with increased volumes of media are necessary to gain more precise and reproducible data.

Due to the outcomes within this study regarding gliadin and ATI degradation as well as acidification parameters, each tested LAB isolate showed the potential to decrease the immunogenic potential of wheat proteins. ATI degradation showed minor differences among the used LAB isolates, whereas the degradation of gliadin varied strongly. Overall, *Lp. plantarum* Lpl5 and Lpl7 as well as *Lac. paracasei* Lpl4 exhibited the highest capacity. Similar results were obtained by Huang, et al. [46]. Fermentation by two *F. sanfranciscensis* strains within mini-dough systems revealed ATI degradation of approximately 40% as measured by size-exclusion chromatography. Further measurements of ATI bioactivity showed a strong reduction of cytokine release by different LAB strains compared to whole wheat flour without fermentation [46].

These results will further help in selecting a specific microbiota for the production of bakery products better tolerated by people with NCWS or irritable bowel syndrome. However, further experiments within the matrix sourdough are necessary to determine the effects of all wheat ingredients (e.g., impact of endogenous cereal proteases, sugars modulating growth of microorganism) on the reduction of proteins with adverse effects. Furthermore, synergistic effects between different LAB strains [34,47] need further exploration.

## Figures and Tables

**Table 1:** Acidification parameters and gliadin degradation capacity of LAB after incubation in GBM.

Isolate	Associated Figure	pH	FAN (mg Glycine/L)	Lactic Acid (mg/L)	Acetic Acid (mg/L)	Gliadin Degradation
*Levilactobacillus brevis* Lb3	Figure 2b	6.67	6.77 ± 0.02	114 ± 3	88 ± 2	++
*Latilactobacillus curvatus* Lcu4	Figure 2c	6.55	6.47 ± 0.14	392 ± 49	<LOD	+
*Loigolactobacillus coryniformis* Lco4	Figure 2d	6.65	6.42 ± 0.27	108 ± 17	39 ± 3	++
*Lactiplantibacillus plantarum* Lpl5	Figure 2e	4.49	5.37 ± 0.25	2786 ± 84	34 ± 1	++
*Lactiplantibacillus plantarum* Lpl7	Figure 2f	4.84	5.58 ± 0.11	2392 ± 8	42 ± 2	++
*Lacticaseibacillus paracasei* Lpa4	Figure 2g	4.77	55.67 ± 0.09	2442 ± 12	58 ± 2	+++
*Leuconostoc pseudomesenteroides* Leps1	Figure 2h	6.64	5.01 ± 0.23	223 ± 13	34 ± 0	+
*Pediococcus pentosaceus* Pp3	Figure 2i	6.67	5.87 ± 0.21	190 ± 7	<LOD	+
*Leuconostoc mesenteroides* Lm3	Figure 2j	6.44	3.85 ± 0.13	650 ± 55	184 ± 15	+

Acid concentrations were measured in duplicate (*n* = 2); results are expressed as mg/L ± standard deviation; LOD (limit of detection) defined as ≤29 mg/L; (+) low degradation; (++) intermediate degradation; (+++) strong degradation based on MALDI-TOF MS spectra interpretation; LAB (lactic acid bacteria); GBM (gluten-based medium); FAN (free amino nitrogen).

**Table 2:** Acidification parameters of LAB within ATI-based medium.

Isolate	pH	Lactic Acid (mg/L)	Acetic Acid (mg/L)	FAN (mg Glycine/L)
*Levilactobacillus brevis* Lb3	6.10	585 ± 137	118 ± 1	23.57 ± 0.85
*Latilactobacillus curvatus* Lcu4	6.52	190 ± 12	101 ± 1	27.67 ± 1.57
*Loigolactobacillus coryniformis* Lco4	4.44	1189 ± 38	101 ± 1	8.01 ± 0.78
*Lactiplantibacillus plantarum* Lpl5	4.54	1108 ± 7	103 ± 1	25.39 ± 0.46
*Lactiplantibacillus plantarum* Lpl7	4.12	>1250	102 ± 9	20.50 ± 0.47
*Lacticaseibacillus paracasei* Lpa4	4.02	>1250	101 ± 1	25.51 ± 2.35
*Leuconostoc pseudomesenteroides* Leps1	4.21	>1250	146 ± 17	23.76 ± 3.41
*Pediococcus pentosaceus* Pp3	6.68	178 ± 1	95 ± 4	43.02 ± 1.44
*Leuconostoc mesenteroides* Lm3	6.59	274 ± 87	128 ± 17	32.21 ± 1.79
*Fructilactobacillus sanfranciscensis* Lsa3	6.71	<35	93 ± 1	33.21 ± 5.73

Acid and FAN concentrations were measured in duplicate (*n* = 2); results are expressed as mg/L; ±standard deviation; LAB (lactic acid bacteria); ATI (amylase-trypsin inhibitor).

## Supplementary Materials

The following are available online at https://www.mdpi.com/2076-2607/8/11/1689/s1, Table S1: Growth evaluation on gluten-based medium of 87 lactic acid bacterial isolates, used code within this study, biological origin, and provider, Table S2: Detected and identified proteins in ATI isolate via peptide mass fingerprint and MASCOT search, Figure S1: MALDI-TOF MS analysis in the range of 5–70 kDa of the intermediate of chloroform methanol (CM) extraction (a), the final extract (ATI isolate) (b), and the alpha-amylase inhibitor (AAI) standard from Sigma-Aldrich (c), Figure S2: Example of growth of lactic acid bacteria on gluten-based medium (GBM); *Lacticaseibacillus paracasei* Lpa2, Lpa5, Lpa6; *Pediococcus pentosaceus* Pp2, Pp5; *Leuconostoc citreum* Lec1; isolates were analyzed in duplicate, Figure S3: MALDI-TOF MS spectra of water-soluble peptides in the range of 500 to 4000 of (a) reference, and after the degradation (72 h) by (b) *Levilactobacillus brevis* Lb3, (c) *Latilactobacillus curvatus* Lcu4, (d) *Loigolactobacillus coryniformis* Lco4, (e) *Lactiplantibacillus plantarum* Lpl5, (f) *Lactiplantibacillus plantarum* Lpl7, (g) *Lacticaseibacillus paracasei* Lpa4, (h) *Leuconostoc. pseudomesenteroides* Leps1, (i) *Pediococcus pentosaceus* Pp3, (j) *Leuconostoc mesenteroides* Lm3.
