# Supplementary material for: Insights into the Potential of Sourdough-Related Lactic Acid Bacteria to Degrade Proteins in Wheat
Source: Microorganisms. 2020 Oct 30;8(11):1689. doi: 10.3390/microorganisms8111689 (PMC7693696; doi:10.3390/microorganisms8111689)
Supplement: Supplementary file 1 [file microorganisms-08-01689-s001.pdf]

microorganisms

MDPI

Supplementary material

Table S1. Growth evaluation on gluten-based medium of 87 lactic acid bacterial isolates, used code within this study, biological origin, and provider

Code	Biological origin	Provider	Genus	Species	Growth evaluation
LMG 22083	traditional dairy fermented products	LMG[1]	*Levilactobacillus*	*brevis*	+
Lb1	wheat sourdough	bakery	*Levilactobacillus*	*brevis*	+
Lb2	wheat sourdough	bakery	*Levilactobacillus*	*brevis*	+
Lb3	Wheat sourdough	Bakery	*Levilactobacillus*	*brevis*	++
Lco1	wheat sourdough	bakery	*Loigolactobacillus*	*coryniformis*	++
Lco2	wheat sourdough	bakery	*Loigolactobacillus*	*coryniformis*	+
Lco3	wheat sourdough	bakery	*Loigolactobacillus*	*coryniformis*	++
Lco4	wheat sourdough	bakery	*Loigolactobacillus*	*coryniformis*	++
Lcu1	wheat sourdough	bakery	*Latilactobacillus*	*curvatus*	+
Lcu2	wheat sourdough	bakery	*Latilactobacillus*	*curvatus*	+
Lcu3	wheat sourdough	bakery	*Latilactobacillus*	*curvatus*	+
Lcu4	wheat sourdough	bakery	*Latilactobacillus*	*curvatus*	++
Ld1	rye sourdough	household	*Lentilactobacillus*	*diolivorans*	+
Lg1	rye sourdough	bakery	*Lactobacillus*	*gallinarum*	+
LMG 6906	Human, faeces	LMG	*Levilactobacillus*	*hammesii*	+
DSM 16381	wheat sourdough	DSMZ[2]	*Levilactobacillus*	*hammesii*	+
Lh1	rye sourdough	household	*Levilactobacillus*	*hammesii*	+
LMG 19822	kimchi, a Korean fermented-vegetable food	LMG	*Companilactobacillus*	*kimchii*	+
Lk1	rye sourdough	household	*Companilactobacillus*	*kimchii*	+
DSM 19906	non-salted pickle solution used in production of sunki	DSMZ	*Lentilactobacillus*	*kisonensis*	+
Lki1	rye sourdough	household	*Lentilactobacillus*	*kisonensis*	+
DSM 19908	non-salted pickle solution used in production of sunki	DSMZ	*Lentilactobacillus*	*otakiensis*	-
Lo1	rye sourdough	household	*Lentilactobacillus*	*otakiensis*	+
Lpb1	wheat sourdough	bakery	*Lentilactobacillus*	*parabuchneri*	+
Lpb2	wheat sourdough	bakery	*Lentilactobacillus*	*parabuchneri*	+
Lpb3	wheat sourdough	bakery	*Lentilactobacillus*	*parabuchneri*	-
Lpb4	wheat sourdough	bakery	*Lentilactobacillus*	*parabuchneri*	+
DSM 20258	pasteurized milk	DSMZ	*Lacticaseibacillus*	*paracasei*	+
DSM 5622	unknown	DSMZ	*Lacticaseibacillus*	*paracasei*	+
Lpa1	rye sourdough	bakery	*Lacticaseibacillus*	*paracasei*	++
Lpa2	wheat sourdough	bakery	*Lacticaseibacillus*	*paracasei*	+
Lpa3	wheat sourdough	bakery	*Lacticaseibacillus*	*paracasei*	++
Lpa4	wheat sourdough	bakery	*Lacticaseibacillus*	*paracasei*	++

Code	Biological origin	Provider	Genus	Species	Growth evaluation
Lpa5	wheat sourdough	bakery	*Lacticaseibacillus*	*paracasei*	++
Lpa6	wheat sourdough	bakery	*Lacticaseibacillus*	*paracasei*	+
Lpa7	wheat sourdough	bakery	*Lacticaseibacillus*	*paracasei*	+
Lpa8	rye sourdough	bakery	*Lacticaseibacillus*	*paracasei*	+
LMG 19152	sourdough	LMG	*Companilactobacillus*	*paralimentarius*	+
Lpar1	rye sourdough	household	*Companilactobacillus*	*paralimentarius*	+
Lpar2	rye sourdough	household	*Companilactobacillus*	*paralimentarius*	+
DSM 10667	beer contaminant	DSMZ	*Lactiplantibacillus*	*paraplantarum*	++
Lpl1	wheat sourdough	bakery	*Lactiplantibacillus*	*plantarum*	++
Lpl2	wheat sourdough	bakery	*Lactiplantibacillus*	*plantarum*	++
Lpl3	wheat sourdough	household	*Lactiplantibacillus*	*plantarum*	+
Lpl4	rye sourdough	bakery	*Lactiplantibacillus*	*plantarum*	+
Lpl5	wheat sourdough	bakery	*Lactiplantibacillus*	*plantarum*	++
Lpl6	wheat sourdough	bakery	*Lactiplantibacillus*	*plantarum*	+
Lpl7	wheat sourdough	bakery	*Lactiplantibacillus*	*plantarum*	++
DSM 8475	rye sourdough	DSMZ	*Limosilactobacillus*	*pontis*	+
Lpo1	rye sourdough	bakery	*Limosilactobacillus*	*pontis*	-
Lpo2	wheat sourdough	bakery	*Limosilactobacillus*	*pontis*	+
LMG 22972	wheat sourdough	LMG	*Furfurilactobacillus*	*rossiae*	+
Lr1	rye sourdough	bakery	*Furfurilactobacillus*	*rossiae*	+
Lr2	rye sourdough	bakery	*Furfurilactobacillus*	*rossiae*	+
Lr3	wheat sourdough	bakery	*Furfurilactobacillus*	*rossiae*	+
Lr4	wheat sourdough	bakery	*Furfurilactobacillus*	*rossiae*	+
DSM 20663	sourdough	DSMZ	*Fructilactobacillus*	*sanfranciscensis*	-
Lsa1	rye sourdough	bakery	*Fructilactobacillus*	*sanfranciscensis*	+
Lsa2	wheat sourdough	household	*Fructilactobacillus*	*sanfranciscensis*	+
Lsa3	wheat sourdough	bakery	*Fructilactobacillus*	*sanfranciscensis*	+
LMG 29106	senmaizuke	LMG	*Levilactobacillus*	*senmaizukei*	-
Lse2	wheat sourdough	bakery	*Levilactobacillus*	*senmaizukei*	+
Lse3	wheat sourdough	bakery	*Levilactobacillus*	*senmaizukei*	+
Lse4	wheat sourdough	bakery	*Levilactobacillus*	*senmaizukei*	+
LMG 21871	rice sourdough	LMG	*Levilactobacillus*	*spicheri*	-
Lsp1	rye sourdough	household	*Levilactobacillus*	*spicheri*	+
Lsp2	wheat sourdough	bakery	*Levilactobacillus*	*spicheri*	+
Lsp3	wheat sourdough	bakery	*Levilactobacillus*	*spicheri*	+
Lv1	wheat sourdough	bakery	*Paucilactobacillus*	*vaccinostercus*	+
Lx1	rye sourdough	household	*Lactiplantibacillus*	*xiangfangensis*	+
Lec1	wheat sourdough	bakery	*Leuconostoc*	*citreum*	+
Lm1	wheat sourdough	bakery	*Leuconostoc*	*mesenteroides*	++
Lm2	wheat sourdough	bakery	*Leuconostoc*	*mesenteroides*	+
Lm3	wheat sourdough	bakery	*Leuconostoc*	*mesenteroides*	++
Leps1	wheat sourdough	bakery	*Leuconostoc*	*pseudomesenteroides*	++
Leps2	wheat sourdough	bakery	*Leuconostoc*	*pseudomesenteroides*	++
Leps3	wheat sourdough	bakery	*Leuconostoc*	*pseudomesenteroides*	++
Pp1	wheat sourdough	bakery	*Pediococcus*	*pentosaceus*	+

Code	Biological origin	Provider	Genus	Species	Growth evaluation
Pp2	wheat sourdough	bakery	*Pediococcus*	*pentosaceus*	+
Pp3	wheat sourdough	bakery	*Pediococcus*	*pentosaceus*	++
Pp4	wheat sourdough	bakery	*Pediococcus*	*pentosaceus*	+
Pp5	wheat sourdough	bakery	*Pediococcus*	*pentosaceus*	+
Pp6	wheat sourdough	bakery	*Pediococcus*	*pentosaceus*	+
Pp7	wheat sourdough	bakery	*Pediococcus*	*pentosaceus*	+
LMG 17699	chili bo	LMG	*Weissella*	*cibaria*	+
LMG 27187	sourdough	LMG	*Weissella*	*cibaria*	+
Wv1	wheat sourdough	bakery	*Weissella*	*viridescens*	+
LMG 3507	Cured meat products	LMG	*Weissella*	*viridescens*	+

Table S2. Detected and identified proteins in ATI isolate via peptide mass fingerprints and MASCOT search.

Accession	Name	MASCOT Score	Peptides matched	SC [%]
IAA1_WHEAT	**Alpha-amylase inhibitor 0.19** OS=Triticum aestivum PE=1 SV=1	412.0	57	67.7
IAA5_WHEAT	**Alpha-amylase inhibitor 0.53** OS=Triticum aestivum PE=1 SV=1	395.5	55	58.1
IAAC3_WHEAT	**Alpha-amylase/trypsin inhibitor CM3** OS=Triticum aestivum PE=1 SV=1	318.7	14	52.4
IAA2_WHEAT	**Alpha-amylase inhibitor 0.28** OS=Triticum aestivum GN=IMA1 PE=1 SV=3	276.0	8	43.8
IBB3_WHEAT	**Bowman-Birk type trypsin inhibitor** OS=Triticum aestivum PE=1 SV=1	170.9	9	74.6
IAC16_WHEAT	**Alpha-amylase/trypsin inhibitor CM16** OS=Triticum aestivum PE=1 SV=1	122.3	5	39.2
IAAD_HORVU	**Alpha-amylase/trypsin inhibitor CMd** OS=Hordeum vulgare GN=IAT3 PE=1 SV=2	115.6	5	18.7
IBB1_WHEAT	**Bowman-Birk type proteinase inhibitor I-2B (Fragment)** OS=Triticum aestivum PE=1 SV=1	113.8	4	58.9
NLTP1_WHEAT	**Non-specific lipid-transfer protein (Fragment)** OS=Triticum aestivum PE=1 SV=2	112.7	4	31.0
RIP2_HORVU	**Protein synthesis inhibitor II** OS=Hordeum vulgare GN=RIP30A PE=1 SV=1	109.2	3	11.1
AVLA2_WHEAT	**Avenin-like a2** OS=Triticum aestivum PE=2 SV=1	107.0	3	22.3
NLT2G_WHEAT	**Non-specific lipid-transfer protein 2G** OS=Triticum aestivum PE=1 SV=2	101.2	5	34.4
NLT2P_WHEAT	**Non-specific lipid-transfer protein 2P** OS=Triticum aestivum PE=1 SV=1	98.1	5	32.8
AVLA4_WHEAT	**Avenin-like a4** OS=Triticum aestivum PE=2 SV=1	96.9	3	26.2

IAAC2_WHEAT	**Alpha-amylase/trypsin inhibitor CM2** OS=Triticum aestivum PE=1 SV=2	94.6	4	24.8
AVLB8_WHEAT	**Avenin-like b8** OS=Triticum aestivum PE=3 SV=1	92.4	2	7.7
AVLA1_WHEAT	**Avenin-like a1** OS=Triticum aestivum GN=AVNLA PE=2 SV=1	86.3	3	20.8
THN2_WHEAT	**Alpha-2-purothionin** OS=Triticum aestivum GN=THI1.2 PE=2 SV=1	78.8	4	16.9
THNB_WHEAT	**Purothionin A-1** OS=Triticum aestivum GN=THI1.3 PE=1 SV=2	76.8	4	16.9
ICIC_HORVU	**Subtilisin-chymotrypsin inhibitor CI-1C** OS=Hordeum vulgare PE=1 SV=1	59.4	2	16.9
MPAP6_PHLPR	Pollen allergen Phl p 6 OS=Phleum pratense GN=PHLPVI PE=1 SV=1	51.5	9	18.2
TRY1_BOVIN	Cationic trypsin OS=Bos taurus PE=1 SV=3	239	9	39.8
TRYP_PIG	Trypsin OS=Sus scrofa PE=1 SV=1	150.7	8	16.5
AOC1_HUMAN	Amiloride-sensitive amine oxidase [copper-containing] OS=Homo sapiens GN=AOC1 PE=1 SV=4	126.8	40	7.7
BARW_HORVU	Barwin OS=Hordeum vulgare PE=1 SV=1	70.4	3	26.4
IAAS_WHEAT	Endogenous alpha-amylase/subtilisin inhibitor OS=Triticum aestivum PE=1 SV=1	53.4	1	7.2
CHIC_SECCE	Basic endochitinase C OS=Secale cereale GN=rscc PE=1 SV=1	48.8	1	5.3
LE19A_HORVU	Late embryogenesis abundant protein B19.1A OS=Hordeum vulgare GN=B19.1A PE=2 SV=1	42.1	1	12.9
MT1A_MORBO	Modification methylase MboIA OS=Moraxella bovis GN=mboIAM PE=3 SV=1	41.6	1	2
CUCIN_ORYSI	Cupincin OS=Oryza sativa subsp. indica GN=OsI_13867 PE=1 SV=1	38.9	2	2.1
AZOR_GLUOX	FMN-dependent NADH-azoreductase OS=Gluconobacter oxydans (strain 621H) GN=azoR PE=3 SV=1	36.2	1	3.5
THN1_WHEAT	Alpha-1-purothionin (Fragment) OS=Triticum aestivum GN=THI1.1 PE=1 SV=2	35.3	2	11.1
AMYB_HORVS	Beta-amylase OS=Hordeum vulgare subsp. spontaneum GN=BMY1 PE=1 SV=1	34.5	1	2.8
THHR_HORVU	Antifungal protein R (Fragment) OS=Hordeum vulgare PE=1 SV=1	32.6	2	18.2
LEUC_MYCGI	3-isopropylmalate dehydratase large subunit OS=Mycobacterium gilvum (strain PYR-GCK) GN=leuC PE=3 SV=1	32.2	1	1.9

NLTPB_WHEAT	Probable non-specific lipid-transfer protein (Fragment) OS=Triticum aestivum PE=1 SV=1	30.5	1	55.0

Clearly identified proteins are in bold letters (number of identified peptides higher than 2 and MASCOT score higher than 55); impurities from other sources than cereals (e.g. Amiloride-sensitive amine oxidase from humans) and residues from digestion (trypsin) were not assigned as identified proteins.

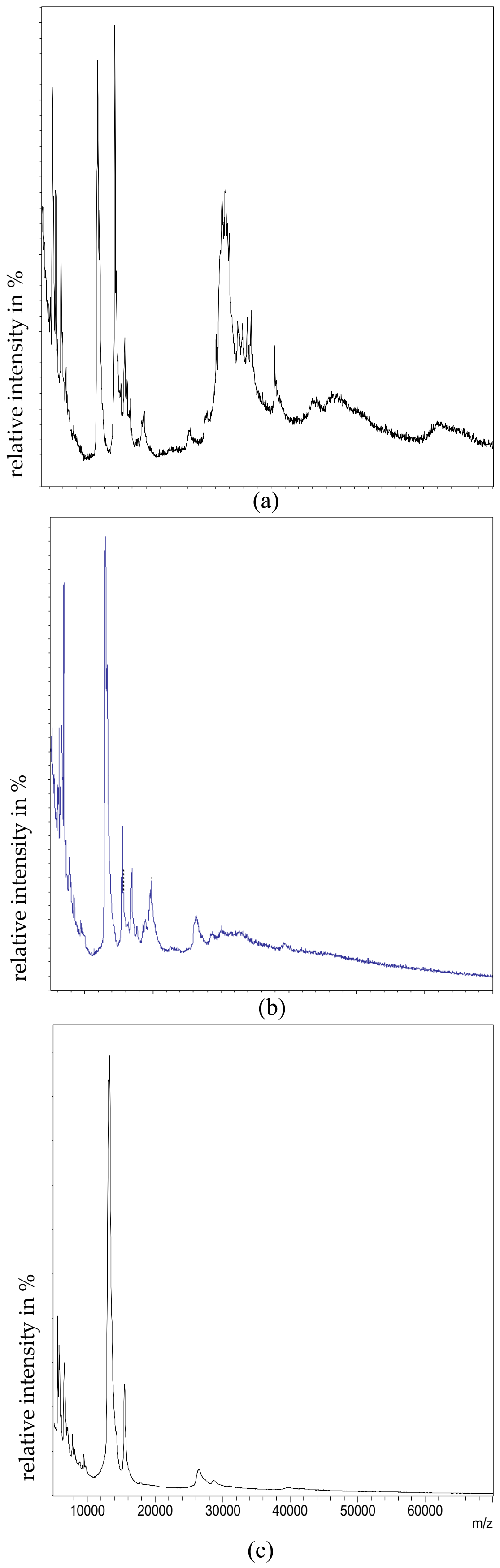

Figure S1. MALDI-TOF MS analysis in the range from 5 – 70 kDa of the intermediate of chloroform methanol (CM) extraction (a), the final extract (ATI isolate) (b), and the alpha-amylase inhibitor (AAI) standard (c) (Sigma-Aldrich, USA)

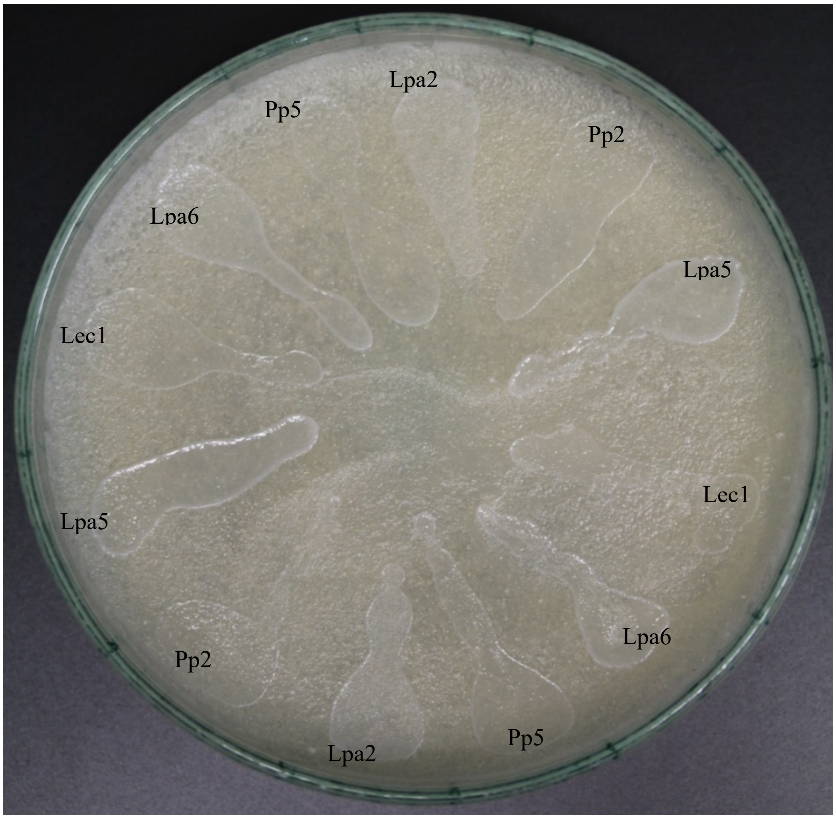

Figure S2. Example for growth of lactic acid bacteria on gluten-based medium (GBM); *Lacticaseibacillus paracasei* Lpa2, Lpa5, Lpa6; *Pediococcus pentosaceus* Pp2, Pp5; *Leuconostoc citreum* Lec1; Isolates were analysed in duplicate;

14

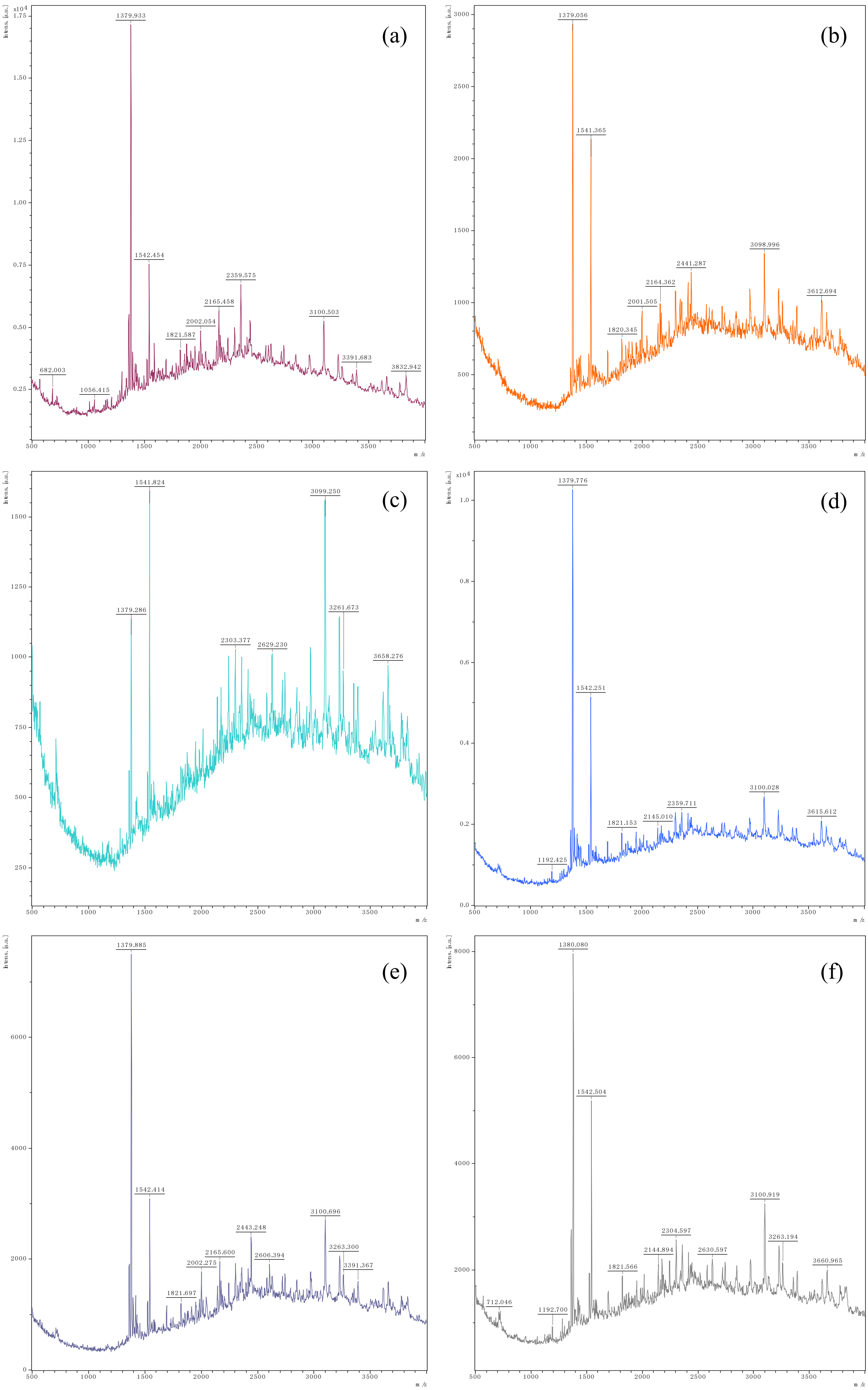
(a)
Intens. [a.u.]
x10^4
1379.933
1542.454
2359.575
2165.458
2002.054
1821.587
3100.503
3391.683
3832.942
682.003
1056.415
m/z
(b)
1379.056
1541.365
3098.996
2441.287
2164.362
2001.505
1820.345
3612.694
(c)
1541.824
3099.250
1379.286
3261.673
2303.377
2629.230
3658.276
(d)
1379.776
1542.251
3100.028
2359.711
1821.153
2145.010
3615.612
1192.425
(e)
1379.885
1542.414
3100.696
2443.248
2165.600
3263.300
2002.275
2606.394
3391.367
1821.697
(f)
1380.080
1542.504
3100.919
2304.597
3263.194
2144.894
2630.597
1821.566
3660.965
712.046
1192.700

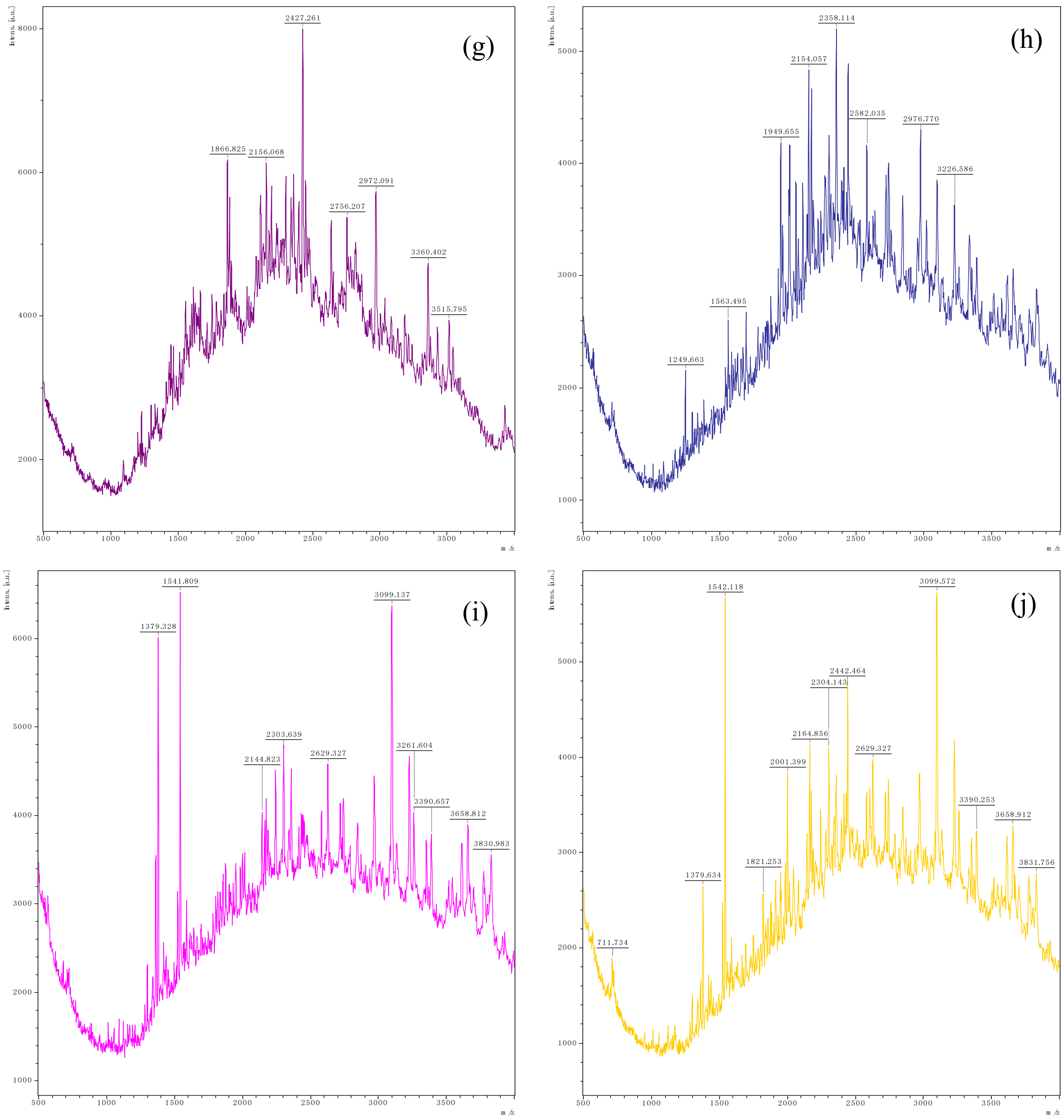

Figure S3. MALDI-TOF MS spectra of water-soluble peptides in the range of 500 to 4000 of (a) Reference, and after the degradation (72 h) by (b) *Levilactobacillus brevis* Lb3, (c) *Latilactobacillus curvatus* Lcu4, (d) *Loigolactobacillus coryniformis* Lco4, (e) *Lactiplantibacillus plantarum* Lpl5, (f) *Lactiplantibacillus plantarum* Lpl7, (g) *Lacticaseibacillus paracasei* Lpa4, (h) *Leuconostoc pseudomesenteroides* Leps1, (i) *Pediococcus pentosaceus* Pp3, (j) *Leuconostoc mesenteroides* Lm3
